# *H19* gene polymorphisms and neuroblastoma susceptibility in Chinese children: a six-center case-control study

**DOI:** 10.7150/jca.37564

**Published:** 2019-10-18

**Authors:** Yong Li, Zhen-Jian Zhuo, Haiyan Zhou, Jiabin Liu, Jiao Zhang, Jiwen Cheng, Haixia Zhou, Suhong Li, Ming Li, Jun He, Zhenghui Xiao, Jing He, Yaling Xiao

**Affiliations:** 1 Department of Pediatric Surgery, Hunan Children's Hospital, Changsha 410004, Hunan, China.; 2 Department of Pediatric Surgery, Guangzhou Institute of Pediatrics, Guangdong Provincial Key Laboratory of Research in Structural Birth Defect Disease, Guangzhou Women and Children's Medical Center, Guangzhou Medical University, Guangzhou 510623, Guangdong, China.; 3 Department of Pathology, Xiang-ya School of Medicine, Central South University, Changsha 410013, Hunan, China.; 4 Department of Pediatric Surgery, the First Affiliated Hospital of Zhengzhou University, Zhengzhou 450052, Henan, China.; 5 Department of Pediatric Surgery, the Second Affiliated Hospital of Xi'an Jiaotong University, Xi'an 710004, Shaanxi, China.; 6 Department of Hematology, The Second Affiliated Hospital and Yuying Children's Hospital of Wenzhou Medical University, Wenzhou 325027, Zhejiang, China.; 7 Department of Pathology, Children Hospital and Women Health Center of Shanxi, Taiyuan 030013, Shannxi, China.; 8 Emergency center of Hunan Children's Hospital, Changsha 410004, Hunan, China.

**Keywords:** lncRNA, *H19*, polymorphism, neuroblastoma, susceptibility

## Abstract

Neuroblastoma is the most common seen solid tumor in children less than one year old. Given that polymorphisms in the lncRNA* H19* gene are observed in several types of human malignancies, there likely to be similar events that contribute to the pathogenesis of neuroblastoma. We hypothesize that single nucleotide polymorphisms (SNPs) in the *H19* gene might predispose to neuroblastoma. Here, we genotyped three SNPs (rs2839698 G>A, rs3024270 C>G, rs217727 G>A) from *H19* gene in a Chinese population (700 subjects with neuroblastoma and 1516 control subjects) enrolled from six hospitals and examined the effect of individual and combined SNPs on the risk of neuroblastoma. Odds ratios (ORs) and 95% confidence intervals (CIs) calculated from logistic regression were adopted to assess such association, adjusted for age and gender. Among them, 700 controls and 1514 cases were successfully genotyped. None of these three SNPs were found to be relevant to the risk of neuroblastoma, either in overall analysis or stratification analysis. Findings from this study excluded the participation of lncRNA *H19* gene SNPs in the risk of neuroblastoma. More independent case-control studies are encouraged to better elucidate this relationship.

## Introduction

Neuroblastoma is a childhood tumor that mainly derives from neural crest progenitor cells 1, 2. Despite its occupation rate of 8-10% of all pediatric cancer diagnoses, neuroblastoma disproportionately results in 12-15% of all childhood cancer related mortality 3-5. It is characterized by widely clinical heterogeneity, spanning from spontaneous regression to therapy-refractory progression 6, 7. Another reflection of such heterogeneity was the contrasting survival rate of different subgroup patients 8. In patients with the low- and intermediate-risk neuroblastoma, the long-term survival rate is greater than 90%. However, in patients with the high-risk neuroblastoma, less than 40% could finally survived 9-12.

In the past decades, considerable progress has been made in understanding the genetic underpinnings of neuroblastoma. Exposed environmental factors of children and pregnant women were reported to predispose to neuroblastoma, but not finally defined 13, 14. Mutations in *ALK* and *PHOX2B* were considered as two major causes of familial neuroblastoma. Other genetic variants in genes including* LMO1* 15,* BARD1* 16,* TP53* 17, *DUSP12* 18, *LIN28B* 19, *HACE1* 19, *NEFL* 20 and *CDKN1B* 21 have more recently been associated with neuroblastoma predisposition. Moreover, fine mapping study also identified neuroblastoma-associated functional risk SNPs in *BARD1* 22. Taken together, however, all the current identified variants still could not fully elucidate the etiology of neuroblastoma. We still have a long way to fully reveal the genetic landscape of neuroblastoma. Identification of other somatic mutations and polymorphisms will further clarify the mechanisms of neuroblastoma.

Long non-coding RNAs (lncRNAs), longer than 200 nucleotides, are a class of non-protein-coding transcripts. LncRNAs regulate multiple physiological and pathological processes by different mechanisms, including transcriptional regulation, genome rearrangement, imprinting, epigenetic regulation, and chromatin modification. Accumulating evidence point to the critical role of lncRNAs in human disorders especially in cancers. *H19* gene was the first lncRNA discovered in the human genome 23, 24. *H19* gene is exclusively expressed from maternal chromosome, treated as a paternally imprinted oncofetal gene 25. *H19* gene is incompetent to encode for a protein, but instead encodes for a polyadenylated, spliced, and capped ncRNA spans 2.7kb long 23. *H19* gene has been reported as a cancer-associated lncRNA that functions as both oncogene and suppressor gene. Mounting proof have been achieved in the modulation ability of single nucleotide polymorphisms (SNPs) within lncRNAs in causing tumor susceptibility. Thus far, most studies have addressed the identification of *H19* gene SNPs in bladder cancer 26, hepatocellular cancer 27, and lung cancer 28, whereas few studies focused on the role of *H19* gene SNPs in neuroblastoma risk. Here we conducted a six-center hospital-based case-control study aiming to investigate the association between SNPs within the *H19* gene and neuroblastoma risk.

## Materials and Methods

### Study subjects

The enrollment criteria of subjects were described in previous studies 29-32. Prior to analysis, the study protocols were approved by the Institutional Review Board of each hospital. The current case-control study was carried out in 6 hospitals in 6 provinces of China. A total of 700 cases were pathology-confirmed with neuroblastoma, and classified clinical stages according to the INSS 33, and 1516 controls with no prior history of neuroblastoma were randomly enrolled in the same area as cases. To be specific, 275 cases and 531 controls were from Guangzhou Women and Children's Medical Center 34, 162 cases and 270 controls were from Hunan Children's Hospital 35, 118 cases and 281 controls were from The First Affiliated Hospital of Zhengzhou University 36, 37, 76 cases and 186 controls were from The Second Affiliated Hospital of Xi'an Jiaotong University 38, 36 cases and 72 controls were from The Second Affiliated Hospital and Yuying Children's Hospital of Wenzhou Medical University 39, 40, and 33 cases and 176 controls were from Children's Hospital of Shanxi. All guardians of participants provided written informed consent.

### Polymorphism selection and genotyping

We screened NCBI dbSNP database (http://www.ncbi.nlm.nih.gov/projects/SNP) and SNPinfo (http://snpinfo.niehs.nih.gov/snpfunc.htm) to select potential functional neuroblastoma-risk SNPs. Three SNPs (rs2839698 G>A, rs3024270 C>G, rs217727 G>A) in *H19* gene that met the previously described criteria were selected for inclusion 41. The rs2839698 G>A and rs3024270 C>G are located in the transcription factor binding sites. As shown in Supplemental Figure s1, there was no significant linkage disequilibrium (R^2^<0.8) among these three SNPs (R^2^=0.408 between rs217727 and rs3024270; R^2^=0.213 between rs217727 and rs2839698; R^2^=0.508 between rs3024270 and rs2839698). Genomic DNA was isolated from venous blood using a TIANamp Blood DNA Kit (TianGen Biotech Co. Ltd., Beijing, China). Genotype analysis of all three *H19* gene SNPs was undertaken using TaqMan SNP Genotyping Assay from Applied Biosystem using 7900 Real-Time PCR system 42-45. Negative controls (with water) and duplicate test samples (10% of all the samples) were included in each 384-well plate. 100% concordant of genotypes in replicates were achieved.

### Statistical analysis

Tests for Hardy-Weinberg equilibrium (HWE) were conducted for each SNP separately among control subjects with the use of χ^2^ test. Differences in demographic variables between case subjects and control subjects were analyzed using the two-sided χ^2^ test. Neuroblastoma risk was determined as odds ratios (ORs) and 95% confidence intervals (CIs), based on unconditional logistic regression adjusted for age and gender. A *P*-value of < 0.05 was used for statistical significance. The SAS release 9.1 (SAS Institute, Cary, NC) was used for statistical analyses.

## Results

### Association between *H19* polymorphisms and neuroblastoma susceptibility

We enrolled 700 cases with neuroblastoma and 1516 controls without neuroblastoma from 6 hospitals in 6 provinces of China (Supplemental Table 1), and no significant differences were observed between cases and controls for age (*P*=0.525) and gender (*P*=0.796). We successfully genotyped 700 controls and 1514 cases. The genotype distributions of *H19* gene polymorphisms (rs2839698 G>A, rs3024270 C>G, rs217727 G>A) and their associations with neuroblastoma susceptibility were summarized in Table 1. In the controls, the genotype frequencies of all the three SNPs conformed to Hardy-Weinberg equilibrium (HWE=0.406 for rs2839698 G>A, HWE=0.162 for rs3024270 C>G, HWE=0.744 for rs217727 G>A). Our results indicated that none of the SNPs included were significantly associated with neuroblastoma susceptibility. We also failed to find significantly statistical correlation between the combined effect of risk genotypes and the susceptibility of neuroblastoma.

### Stratification analysis

We further carried out stratified analysis to analyze the association between* H19* gene polymorphisms and neuroblastoma susceptibility under different subtypes including age, gender, tumor sites, and INSS stages (Table 2). However, we still could not obtain significant association between all the three polymorphisms, neither in single nor combined analysis, and neuroblastoma susceptibility under all the strata.

### Haplotype analysis

As shown in Table 3, we failed to find any haplotype that was significantly associated with neuroblastoma risk.

## Discussion

In this study, we investigated whether lncRNA *H19* gene polymorphisms were involved in the risk of neuroblastoma in Chinese population using individuals from six centers. Our results showed that none of the *H19* gene polymorphisms contributed to the risk of neuroblastoma. Conclusions obtained here represent an important extension of our understanding of how *H19* gene polymorphism is not relevant to neuroblastoma risk.

The* H19* gene is located in human chromosome 11p15.5 locus. The expression level of *H19* gene peaks at the early stages of embryogenesis, then falls postnatally 46, 47, and is reactivated during tumorigenesis in some cancer types 48, 49. The critical role of *H19* gene in carcinogenesis was intensively reported. Wada et al. 50 detected a maintenance of normal imprinting of the *H19* gene in neuroblastoma. Liang et al. 51 found that the lncRNA *H19* gene promotes epithelial to mesenchymal transition of colorectal cancer by functioning as part of miRNA sponges. Barsyte-Lovejoy et al. 52 showed that down regulation of* H19* gene attenuated the clonogenicity of lung cancer and breast cancer. They also provided a strong association between *H19* gene expression and primary lung and breast carcinomas. By now, some SNPs located on *H19* gene were identified to be associated with cancer susceptibility. In a study conducted by Guo et al. 53, it was found that rs217727 AA genotype of *H19* gene contributes to the susceptibility of oral squamous cell carcinoma in Chinese population. Li et al. 28 conducted a case-control study in lung cancer using 555 cases and 618 healthy controls. Their results gave some indication that AA genotype of rs217727 SNP in lncRNA *H19* gene plays a positive role in susceptibility to lung cancer.

We have previously conducted an initial investigation of how *H19* gene SNPs (rs2839698, rs3024270, and rs217727) regulate the risk of neuroblastoma in Chinese population 54. At that time, we only genotyped 393 neuroblastoma patients and 812 control subjects from two hospitals. Our previous results failed to reveal significant associations of the three selected polymorphisms and neuroblastoma susceptibility, in separated and combined analyses. However, we detected an increased neuroblastoma risk in rs3024270 GG genotype in female Chinese children. SNPs analyzed in our previous and the current studies are all located in the *H19* gene. It has been elucidated that these SNPs altered the folding architectures of *H19* gene, which may result in changes of *H19* gene expression or functions 55. Given that lncRNA *H19* gene is critical in carcinogenesis, better understanding is needed of how lncRNA *H19* gene SNPs might impact neuroblastoma risk. Herein, we adopted a larger sample size enrolled from six hospitals in China to get a more generalized conclusion. Again, we failed to detect the impact of *H19* gene SNPs on the risk of neuroblastoma. Moreover, the contributing role of rs3024270 GG in neuroblastoma risk in female Chinese children found in our previous study disappeared in the current larger study. Such conclusion inconsistency may be attributed to the relative compromised statistic power caused by the small sample size in the stratification analysis.

Strength of the current study also accompany some limitations. First, statistic power might be compromised as sample size is not large enough, although this is a multi-center study. Second, as a hospital-based case-control study, inclusion of the non-representative subjects in this study may result in inherent selection bias. Third, conclusions obtained here lack generalizability as subjects are all genetic Chinese descendants. Therefore, cautions should be taken if extrapolated the current conclusion to other populations. Fourth, the selected SNPs were based on prior knowledge of potentially functional SNPs. Other important tagging SNPs within *H19* gene may be omitted. Last, environment factors and gene-environment interactions could not be assessed in the current study, with the absence of environmental data.

In conclusion, here we failed to provide the possibility of lncRNA *H19* gene SNPs in predicting neuroblastoma risk. Our study serves as a basis for future replication studies in independent populations or for functional studies of lncRNA *H19* gene SNPs in neuroblastoma risk.

## Supplementary Material

Supplementary figures and tables.

## Figures and Tables

**Table 1:** Associations between *H19* polymorphisms and neuroblastoma susceptibility

Genotype	Cases (N=700)	Controls (N=1514)	*P*^ a^	Crude OR (95% CI)	*P*	Adjusted OR (95% CI) ^b^	*P*^ b^
rs2839698 G>A (HWE=0.406)							
GG	331 (47.29)	704 (46.50)		1.00		1.00	
AG	300 (42.86)	667 (44.06)		0.96 (0.79-1.16)	0.646	0.96 (0.79-1.16)	0.658
AA	69 (9.86)	143 (9.45)		1.03 (0.75-1.41)	0.871	1.04 (0.76-1.43)	0.805
Additive			0.858	0.99 (0.86-1.14)	0.900	1.00 (0.87-1.14)	0.956
Dominant	369 (52.71)	810 (53.50)	0.730	0.97 (0.81-1.16)	0.730	0.97 (0.81-1.16)	0.762
Recessive	631 (90.14)	1371 (90.55)	0.759	1.05 (0.78-1.42)	0.758	1.06 (0.79-1.44)	0.696
rs3024270 C>G (HWE=0.162)							
CC	184 (26.29)	415 (27.41)		1.00		1.00	
CG	362 (51.71)	781 (51.59)		1.05 (0.84-1.29)	0.684	1.05 (0.85-1.30)	0.679
GG	154 (22.00)	318 (21.00)		1.09 (0.84-1.42)	0.505	1.09 (0.84-1.42)	0.505
Additive			0.799	1.05 (0.92-1.19)	0.503	1.05 (0.92-1.19)	0.503
Dominant	516 (73.71)	1099 (72.59)	0.580	1.06 (0.87-1.30)	0.580	1.06 (0.87-1.30)	0.576
Recessive	546 (78.00)	1196 (79.00)	0.595	1.06 (0.85-1.32)	0.595	1.06 (0.85-1.32)	0.598
rs217727 G>A (HWE=0.744)							
GG	331 (47.29)	679 (44.85)		1.00		1.00	
AG	289 (41.29)	674 (44.52)		0.88 (0.73-1.06)	0.187	0.88 (0.72-1.06)	0.172
AA	80 (11.43)	161 (10.63)		1.02 (0.76-1.37)	0.900	1.02 (0.75-1.37)	0.922
Additive			0.359	0.96 (0.84-1.10)	0.591	0.96 (0.84-1.10)	0.561
Dominant	369 (52.71)	835 (55.15)	0.284	0.91 (0.76-1.09)	0.284	0.90 (0.75-1.08)	0.263
Recessive	620 (88.57)	1353 (89.37)	0.577	1.08 (0.82-1.44)	0.577	1.08 (0.81-1.44)	0.586
Combined effect of risk genotypes ^c^							
0	112 (16.00)	258 (17.04)		1.00		1.00	
1	511 (73.00)	1109 (73.25)		1.06 (0.83-1.36)	0.634	1.06 (0.83-1.36)	0.643
2	77 (11.00)	147 (9.71)		1.21 (0.85-1.72)	0.298	1.22 (0.86-1.74)	0.272
1-2	588 (84.00)	1256 (82.96)	0.542	1.08 (0.85-1.37)	0.542	1.08 (0.85-1.38)	0.542

OR, odds ratio; CI, confidence interval; HWE, Hardy-Weinberg equilibrium. ^a^ χ^2^ test for genotype distributions between neuroblastoma patients and cancer-free controls. ^b^ Adjusted for age and gender. ^c^ Risk genotypes were carriers with rs2839698 AA, rs3024270 CG/GG and rs217727 AA genotypes.

**Table 2:** Stratification analysis for association between *H19* genotypes and neuroblastoma susceptibility

Variables	rs2839698 (case/control)		AOR (95% CI)^a^	*P*^a^	rs3024270 (case/control)		AOR (95% CI)^a^	*P*^a^	rs217727 (case/control)		AOR (95% CI)^a^	*P*^a^	Risk genotypes (case/control)		AOR (95% CI)^a^	*P*^a^
	GG/AG	AA			CC	CG/GG			GG/AG	AA			0	1-2		
Age, month																
≤18	243/554	31/61	1.14 (0.72-1.80)	0.589	69/182	205/433	1.25 (0.90-1.72)	0.184	248/538	26/77	0.73 (0.46-1.17)	0.189	44/107	230/508	1.09 (0.74-1.60)	0.662
>18	388/817	38/82	0.97 (0.65-1.45)	0.880	115/233	311/666	0.95 (0.73-1.23)	0.679	372/815	54/84	1.40 (0.98-2.02)	0.068	68/151	358/748	1.06 (0.78-1.45)	0.703
Gender																
Female	273/600	34/55	1.37 (0.87-2.15)	0.177	76/188	231/467	1.22 (0.90-1.67)	0.206	274/581	33/74	0.93 (0.60-1.44)	0.744	49/117	258/538	1.13 (0.78-1.63)	0.520
Male	358/771	35/88	0.87 (0.57-1.31)	0.490	108/227	285/632	0.95 (0.73-1.24)	0.700	346/772	47/87	1.21 (0.83-1.76)	0.325	63/141	330/718	1.03 (0.75-1.43)	0.848
Sites of origin																
Adrenal gland	196/1371	19/143	0.94 (0.57-1.55)	0.808	58/415	157/1099	1.02 (0.74-1.40)	0.927	185/1353	30/161	1.38 (0.90-2.10)	0.138	34/258	181/1256	1.09 (0.74-1.61)	0.673
Retroperitoneal	219/1371	21/143	0.92 (0.57-1.49)	0.746	61/415	179/1099	1.10 (0.81-1.51)	0.544	217/1353	23/161	0.90 (0.57-1.42)	0.641	38/258	202/1256	1.09 (0.75-1.58)	0.663
Mediastinum	157/1371	20/143	1.24 (0.75-2.04)	0.398	45/415	132/1099	1.12 (0.78-1.60)	0.530	157/1353	20/161	1.06 (0.65-1.74)	0.814	26/258	151/1256	1.21 (0.78-1.87)	0.394
Others	52/1371	8/143	1.44 (0.67-3.10)	0.347	15/415	45/1099	1.14 (0.63-2.07)	0.671	55/1353	5/161	0.76 (0.30-1.92)	0.559	11/258	49/1256	0.92 (0.47-1.79)	0.796
Clinical stages																
I+II+4s	311/1371	34/143	1.05 (0.71-1.55)	0.823	97/415	248/1099	0.97 (0.75-1.26)	0.806	304/1353	41/161	1.13 (0.79-1.63)	0.504	58/258	287/1256	1.02 (0.75-1.39)	0.902
III+IV	297/1371	33/143	1.11 (0.74-1.66)	0.616	80/415	250/1099	1.18 (0.90-1.56)	0.233	294/1353	36/161	1.03 (0.70-1.52)	0.883	50/258	280/1256	1.16 (0.83-1.61)	0.396

AOR, adjusted odds ratio; CI, confidence interval. ^a^ Adjusted for age and gender, omitting the corresponding stratify factor.

**Table 3:** The frequency of inferred haplotypes of *H19* based on observed genotypes and their association with neuroblastoma susceptibility

Haplotypes ^a^	Cases (n=1400)	Controls (n=3028)	Crude OR (95% CI)	*P*	Adjusted OR ^b^ (95% CI)	*P* ^b^
GCG	496 (35.43)	1098 (36.26)	1.00		1.00	
GCA	226 (16.14)	501 (16.55)	1.00 (0.83-1.21)	0.988	1.00 (0.82-1.20)	0.966
GGG	156 (11.14)	303 (10.01)	1.14 (0.91-1.42)	0.245	1.14 (0.91-1.42)	0.259
GGA	84 (6.00)	173 (5.71)	1.08 (0.81-1.42)	0.615	1.07 (0.80-1.41)	0.659
ACG	2 (0.14)	5 (0.17)	0.89 (0.17-4.58)	0.885	0.95 (0.18-4.90)	0.947
ACA	6 (0.43)	7 (0.23)	1.90 (0.63-5.68)	0.252	1.96 (0.65-5.87)	0.230
AGG	297 (21.21)	626 (20.67)	1.05 (0.88-1.25)	0.581	1.05 (0.89-1.25)	0.558
AGA	133 (9.50)	315 (10.40)	0.94 (0.74-1.18)	0.563	0.94 (0.74-1.18)	0.567

OR, odds ratio; CI, confidence interval. ^a^ The haplotypes order were rs2839698, rs3024270, and rs217727. ^b^ Obtained in logistic regression models with adjustment for age and gender.

## Abbreviations

lncRNA: long non-coding RNA
SNP: single nucleotide polymorphism
HWE: Hardy-Weinberg equilibrium
OR: odds ratio
CI: confidence interval
