# Supplementary material for: H19 gene polymorphisms and neuroblastoma susceptibility in Chinese children: a six-center case-control study
Source: J Cancer. 2019 Oct 18;10(25):6358–63. doi: 10.7150/jca.37564 (PMC6856749; doi:10.7150/jca.37564)
Supplement: Supplementary file 1 — Supplementary figures and tables. [file jcav10p6358s1.pdf]

Supplemental Table 1. Frequency distribution of selected characteristics in neuroblastoma cases and cancer-free controls

Variables	Combined subjects					Hunan province				
	Cases (n=700)		Controls (n=1516)		*P* [a]	Cases (n=162)		Controls (n=270)		*P* [a]
	No.	%	No.	%		No.	%	No.	%	
Age range, month	0.00-132.00		0.004-156.00		0.525	0.033-130.00		0.033-101.00		0.322
Mean ± SD	33.17±28.14		30.67±25.20			34.56±30.30		27.81±19.83		
≤18	274	39.14	615	40.57		69	42.59	102	37.78	
>18	426	60.86	901	59.43		93	57.41	168	62.22	
Gender					0.796					0.842
Female	307	43.86	656	43.27		79	48.77	129	47.78	
Male	393	56.14	860	56.73		83	51.23	141	52.22	
INSS stages										
I	216	30.86	/	/		48	29.63	/	/	
II	129	18.43	/	/		22	13.58	/	/	
III	134	19.14	/	/		54	33.33	/	/	
IV	196	28.00	/	/		37	22.84	/	/	
4s	16	2.29	/	/		1	0.62	/	/	
NA	9	1.29	/	/		/	/	/	/	
Sites of origin										
Adrenal gland	215	30.71	/	/		31	19.14	/	/	
Retroperitoneal region	240	34.29	/	/		78	48.15	/	/	
Mediastinum	177	25.29	/	/		36	22.22	/	/	
Other region	60	8.57	/	/		17	10.49	/	/	
NA	8	1.14				/	/	/	/	

SD, standard deviation; NA, not available.

[a] Two-sided χ^2 test comparing distributions between neuroblastoma cases and cancer-free controls.

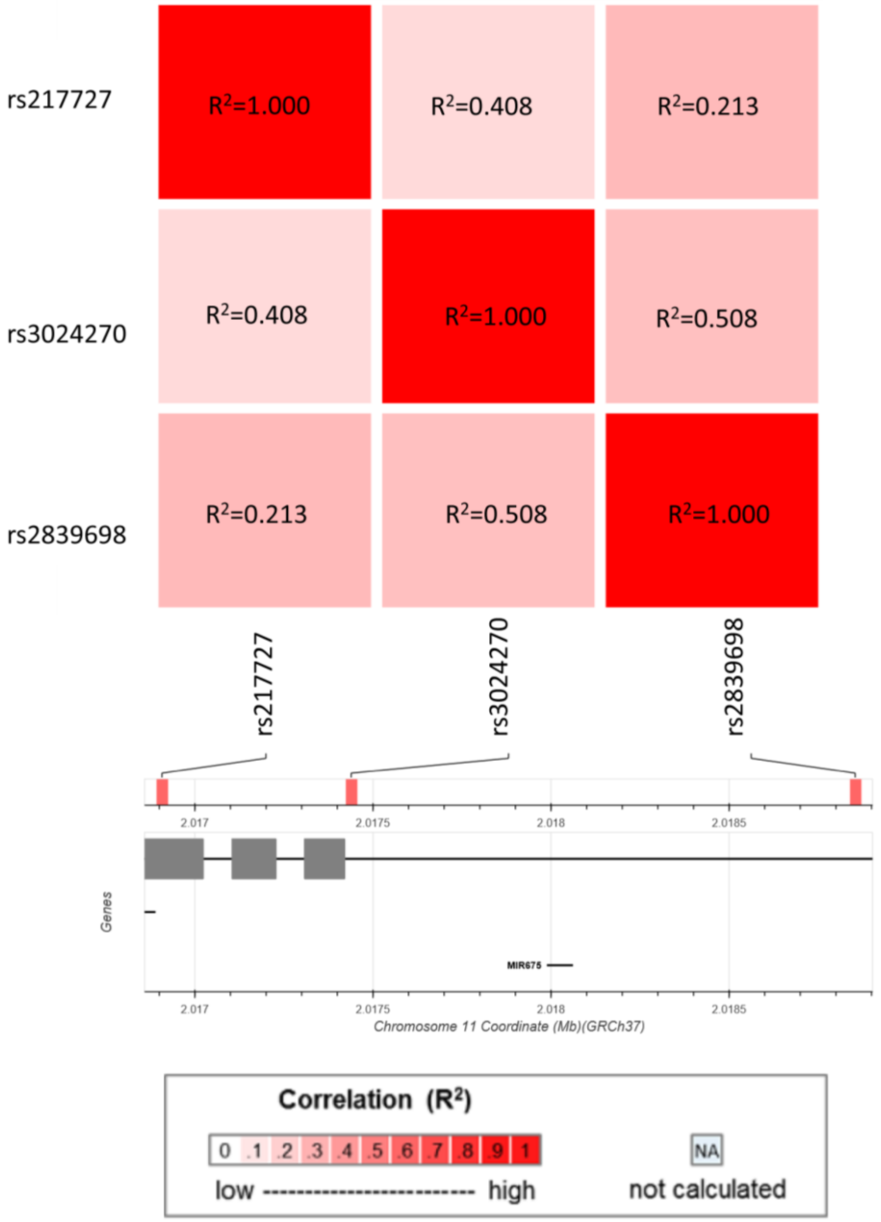

Supplementary Figure 1. Linkage disequilibrium (LD) analysis for the three selected SNPs in Chinese Han population consisting of CHB (Han Chinese in Beijing, China) and CHS (Southern Han Chinese) subjects. LD as R^2 for SNP pairs is shown inside the squares.
